# HBHA induces IL-10 from CD4+ T cells in patients with active tuberculosis but IFN-γ and IL-17 from individuals with *Mycobacterium tuberculosis* infection

**DOI:** 10.3389/fimmu.2024.1422700

**Published:** 2024-08-27

**Authors:** Mai Izumida, Haddijatou Jobe, Edward G. Coker, Amadou Barry, Momodou Rashid, Ismaila L. Manneh, Georgetta K. Daffeh, Koya Ariyoshi, Jayne S. Sutherland

**Affiliations:** ^1^ Vaccines and Immunity Theme, Medical Research Council Unit The Gambia at the London School of Hygiene & Tropical Medicine, Fajara, Gambia; ^2^ Department of Clinical Medicine, Institute of Tropical Medicine, Nagasaki University, Nagasaki, Japan

**Keywords:** *Mycobacterium tuberculosis* (M.tb), TB, LTBI, IL-10, IL-17, IFN-γ, HBHA

## Abstract

**Background:**

To effectively control tuberculosis (TB), it is crucial to distinguish between active TB disease and latent TB infection (LTBI) to provide appropriate treatment. However, no such tests are currently available. Immune responses associated with active TB and LTBI are dynamic and exhibit distinct patterns. Comparing these differences is crucial for developing new diagnostic methods and understanding the etiology of TB. This study aimed to investigate the relationship between pro- and anti-inflammatory CD4+ cytokine production following stimulation with two types of latency-associated *Mycobacterium tuberculosis* (M.tb) antigens to allow differentiation between active TB and LTBI.

**Methods:**

Cryopreserved PBMCs from patients with active TB disease or LTBI were stimulated overnight with replication-related antigen [ESAT-6/CFP-10 (E/C)] or two latency-associated antigens [heparin-binding hemagglutinin (HBHA) and alpha-crystallin-like protein (Acr)]. Responses were analyzed using multiparameter flow cytometry: active TB disease (n=15), LTBI (n=15) and ELISA: active TB disease (n=26) or LTBI (n=27).

**Results:**

CD4+ central memory T cells (Tcm) specific to E/C and CD4+ effector memory T cells specific to Acr and HBHA were higher in LTBI than in TB patients. IFN-γ+Tcm and IL-17+ Tem cells was higher in the LTBI group (p= 0.012 and p=0.029 respectively), but IL-10+ Tcm was higher in the active TB group (p= 0.029) following HBHA stimulation. Additionally, following stimulation with HBHA, IL-10 production from CD4+ T cells was significantly elevated in patients with active TB compared to those with LTBI (*p*= 0.0038), while CD4+ T cell production of IL-17 and IFN-γ was significantly elevated in LTBI compared to active TB (*p*= 0.0076, *p*< 0.0001, respectively). HBHA also induced more CCR6+IL-17+CD4Tcells and IL-17+FoxP3+CD25+CD4Tcells in LTBI than in TB patients (*P*=0.026 and P=0.04, respectively). HBHA also induced higher levels of IFN-γ+IL-10+CD4+ T cells in patients with active TB (Pp=0.03) and higher levels of IFN-γ+IL-17+ CD4+ T cells in those with LTBI (p=0.04). HBHA-specific cytokine production measured using ELISA showed higher levels of IFN-γ in participants with LTBI (P=0.004) and higher levels of IL-10 in those with active TB (P=0.04).

**Conclusion:**

Stimulation with HBHA and measurement of CD4+ T cell production of IFN-γ, IL-10, and IL-17 could potentially differentiate active TB from LTBI. The characteristics of cytokine-expressing cells induced by HBHA also differed between participants with active TB and LTBI.

## Introduction

Tuberculosis (TB) remains the second leading infectious killer after COVID-19. In 2022, TB accounted for 10.6 million new cases and 1.3 million deaths worldwide (1). Approximately 25% of the global population is infected with latent tuberculosis (LTBI), according to a systematic review and meta-analysis of LTBI estimates derived from both interferon-gamma release assays (IGRAs) and tuberculin skin tests (TSTs) (2). Of those with LTBI, 5-10% progress to active TB within two years after the initial exposure (3).

Early detection and treatment of active TB, along with preventive treatment for individuals with LTBI, are crucial for optimal elimination of TB. However, distinguishing between latent and active TB infections is essential to determine the appropriate treatment regimen. Active TB is diagnosed by pathogen detection using methods such as smear microscopy, nucleic acid amplification, or long-term bacterial culture. These methods have limitations, particularly in resource-poor settings, and can misclassify TB cases with no symptoms or low bacterial loads as LTBI (4, 5). While blood-based tests are required to detect LTBI, they currently cannot distinguish between latent and active TB. The World Health Organization (WHO) “End TB Strategy” emphasizes the need for new diagnostic tests that can more accurately diagnose both TB and LTBI (6). The WHO recommends LTBI treatment for individuals at the highest risk of developing active TB (7). However, these guidelines mainly target high- or upper-middle-income countries, which can afford extensive testing and typically have low incidence rates of TB.

Immune responses associated with LTBI and active TB are dynamic and exhibit distinct patterns. Comparing these differences is crucial for developing new diagnostic methods that can distinguish between the two states, and for enhancing our understanding of TB pathogenesis. Furthermore, since the TB immune response varies by ethnicity (8, 9), it is essential to include data from TB endemic and low-income countries.

This study investigated the balance between *Mycobacterium tuberculosis* (M.tb) antigen-specific in clinical samples from participants with active TB or LTBI.

Pro-inflammatory cytokines ‘IFN-γ and IL-17’ and the anti-inflammatory cytokine ‘IL-10’ play crucial roles in the immune response to Mtb, with the balance controlling the overall outcome of the immune response. IL-10 inhibits IFN-γ expression by preventing STAT1 tyrosine phosphorylation (10). IFN-γ, in turn, modifies TLR2-induced signal transduction by increasing GSK3 activity and suppressing MAPK activation, resulting in reduced IL-10 production (11). IL-10 also suppresses the development of Th17 cells, which are involved in inflammation (12).

Previous studies have suggested that cytokine/chemokine profiles induced by specific mycobacterial antigens are promising biomarkers for distinguishing between active TB and LTBI. The latency-associated antigen heparin-binding hemagglutinin (HBHA) is a surface-expressed adhesin of M.tb (13) that has shown potential as an immunodiagnostic test for TB and LTBI. Previous studies in both adult and pediatric cohorts, have reported different cytokines induced by HBHA and ESAT-6. These include IFN-γ and IL-17, to differentiate infected individuals from non-infected individuals and to differentiate patients with active TB from those with LTBI (14–18). In this study we analyzed responses to two latency antigens: recombinant methylated HBHA (19, 20) and Acr (16-kDa antigen), which is a molecular chaperone that is induced during bacteriological persistence and plays a crucial role in the immune response to TB (19, 21). Prior studies from Japan and the Philippines have reported that IL-10 levels produced in response to Acr stimulation differ between active TB and LTBI (22, 23). HBHA has been explored as a potential vaccine component against TB *in vivo* and is used as the fusion protein of the Spore-FP1 vaccine currently under development (13, 24). Analysis of the immune response to HBHA in TB-endemic populations will provide important information for vaccine development.

Previous research has also shown that active TB is associated with higher ratios of effector memory (Tem) to central memory (Tcm) Th1 cells than in LTBI (25, 26). In addition, latency-associated antigens can induce the generation of CD8+Tem during long-term latent Mtb infections (27, 28). We investigated the types of memory CD4+T cells induced by latency-associated antigens in LTBI individuals with a history of high exposure to M.tb, and examined the cytokine responses of these cells.

This study investigated the balance between M.tb antigen-specific cells and soluble markers following stimulation of cells from participants with active TB or LTBI. IL-10, which reduces the pro-inflammatory response, primarily comes from antigen-specific Th1 cells that also produce IFN-γ (29–32). IFN-γ/IL-17 co-expressing CD4-Tcells are also considered important for protective immunity against tuberculosis (33) with active TB patients having a significantly lower proportion of Th17 cells producing both IL-17 and IFN-γ than LTBI subjects (34). Thus, in our study, we aimed to determine whether M. tb antigens could induce double-positive cells associated with TB pathology in both active TB and LTBI contexts. We aimed to verify whether the balance of these cytokine responses differs according to TB infection/disease state in an antigen-specific manner. Using flow cytometry, we compared cytokine-positive CD4+ T cells under different pathological conditions to explore the characteristics of these cells. Additionally, we conducted a pilot study to determine if responses could be detected using ELISA. To understand the pathogenesis of M.tb infection, we also analyzed the characteristics of cytokine-producing cells including effector memory T cells (Tem), central memory T cells (Tcm), CCR6+ T cells, and FoxP3+CD25+ T cells. We investigated the types of memory CD4+T cells induced by latency-associated antigens in LTBI individuals with a history of high exposure to M.tb, and examined the cytokine responses of these cells.

While there are existing reports on immune response differences in blood samples from TB and LTBI patients using latency-associated M.tb antigens, the novelty of this study is that it focuses on cells that produce inflammatory and anti-inflammatory cytokines and analyzes their balance and characteristics. Additionally, we examined the potential of applying this analysis to an ELISA format, considering the ongoing efforts to adapt ELISA for point-of-care (POC) tests. This study is significant as it emerged from a tuberculosis-endemic, low-income country where new assessment systems are needed. To the best of our knowledge, this is the first report in the West African region. Identifying these immunological differences in West Africa can provide valuable insights for the development of new diagnostic or prognostic systems.

## Materials and methods

### Ethics statement

This research was approved by the MRCG/Gambian Government Joint Ethics Committee (SCC1333), and written informed consent was obtained from all participants before sample collection.

### Study participants

Adults with recently diagnosed pulmonary TB were recruited from the Greater Banjul Area in the Gambia in 2016 and 2017. Active pulmonary TB was confirmed by GeneXpert Mtb/RIF assay (Cepheid Inc., Sunnyvale, CA, USA) or sputum liquid culture. LTBI was defined as QuantiFERON TB Gold-in tube positivity (Qiagen, Germany).

Venous blood samples (10ml) were collected using sodium heparin vacutainers (Becton Dickinson, USA). Peripheral blood mononuclear cells (PBMCs) were separated using Lymphoprep in a Leucosep tube (Stem Cell Technologies,USA) and cryopreserved before analysis. All participants included in this study were HIV negative.

### M.tb antigen preparation

ESAT-6/CFP-10, HBHA and Acr expression plasmids were provided by Prof. S. Matsumoto, Niigata University. The coding sequence of the gene with 6X Histidine was initially inserted into the plasmid following the methodology outlined in previous studies (19). Each plasmid was transformed into Clear Coli BL21 (DE3) cells (BIOSEAROLOGIES, USA) by electroporation. Following incubation period in LB broth containing ampicillin, the samples were further incubated in LB broth containing isopropyl-ß-D-thiogalacto-pyranoside. TALON Metal Affinity Resin (TaKaRa, Japan) was used to purify His-tagged proteins. The centrifuged cell culture was resuspended in the equilibration buffer. After centrifugation, the supernatant was transferred into a clean tube and added to the resin. After washing with equilibration buffer, the resin was transferred to gravity-flow column. The His-tagged protein was eluted by adding five bed volumes of Elution Buffer to the column, and SDS-PAGE was used to determine which fraction contained the majority of the His-tagged protein. The technique of purification could not impact the immunogenicity of HBHA as previously shown (19, 22, 35). The optimal concentration of these antigens was determined using existing literature (ESAT-6/CFP-10, Acr and HBHA) (22, 23, 36, 37) and preliminary experiments (Acr and HBHA) (**Supplementary Figure 1**).

### PBMC stimulation

RF10 was prepared with RPMI + 10% FBS + 1% filtered Penicillin/Streptomycin (Sigma Aldrich) + 1% L-glutamine. Cryopreserved PBMCs were thawed in RF10 + 0.02% benzonase in polystyrene tubes at 37°C, 5% CO2. After 6 hours, cells were centrifuged at 500gmax and resuspended at 5.0 × 10^5^ cells in 96 wells with 150μl of RF10. ESAT-6/CFP-10 (0.75μg/ml each), Acr (0.8 μg/ml), mHBHA (1.7μg/ml) (provided by Prof. S. Matsumoto, Niigata University) or NIL (PBS) were added for 16-20 hours. Brefeldin A (5μg/ml, Sigma Aldrich) and monensin (2.5μg/ml, Sigma Aldrich) were added after 4-6 hours of stimulation. After centrifuging at 500gmax for 7 minutes, the supernatant was removed, and cells were incubated with Zombie Aqua (BioLegend, USA) in 100 μl PBS/well for 20 min at room temperature (RT) in the dark. The cells were then washed with PBS and centrifuged. The supernatant was then removed. A cell surface cocktail consisting of anti-human CD3 APC-Cy7, CD4 BUV496, CD69 BV750, CD25 BV786, CD27 PE, CD45RO PE-CY5, CCR6 BB700 and Fc Blocker (BD, USA) was added and incubated for 35 min at 4 °C. The cells were then washed in FACS buffer (PBS, 1% FCS, 0.2% Sodium azide, and 0.1% EDTA), centrifuged, and the supernatant (S/N) was removed. Fixation and Permeabilization Buffer (100 μl) were added (Invitrogen, USA) and incubated for 45 min at 4°C in the dark. After centrifugation, the cells were washed with 100-200 μl of 1× Perm/Wash buffer (Invitrogen, USA) twice. After centrifuging at 550 g for 7 min at 4 °C, S/N was removed, and intracellular staining was performed using IFN-γ AF700, IL-10 BV421, IL-17A/F APC, and FoxP3 PE-CF594 in 1× Perm/Wash buffer, followed by incubation for 45 min at 4 °C in the dark, washing twice, and resuspended in 250μl FACS buffer. The cells were acquired using a FACSymphony flow cytometer (BD Biosciences, USA). The resultant FACS plots were analyzed using FlowJo (Version 10.9.0; Treestar, USA).

### ELISA assay

For IFN-γ and IL-10 ELISA, PBMC were resuspended at 1.0 × 10^5^ cells in 96 wells with 150 μl of RF10. For IL-17 ELISA, PBMC were resuspended at 2.0 × 10^5^ cells in 24 wells with 450 μl of RF10. PBMC were stimulated with PBS (NIL), ESAT-6, CFP-10 (0.75μg/ml each), Acr (0.8 μg/ml), mHBHA (1.7μg/ml), or PMA and ionomycin cocktail (2μl/ml, Invitrogen) for 16 hours. The supernatant was collected into tubes, centrifuged to remove particulates, and stored at -20 °C before analysis. Human IFN-γ (Proteintech, USA), IL-10, and IL-17(R&D Systems) ELISA were performed according to the manufacturer’s protocol. For IFN-γ and IL-10, 20μl of samples and 40 μl of sample were diluted with diluent buffer. For IL-17, 200μl of samples was used per well. A total of 200 μL/well of the sample or standards was incubated overnight at RT.

The plate was washed 3 times with wash buffer. The detection Antibody (100 μL) was added and incubated for 2 hours at RT. After washing, 100 μL streptavidin-HRP was added and incubated for 20 minutes at RT in dark. After washing, 100 μL of Substrate Solution was added and incubated for 20 minutes at RT in the dark. 50 μL of Stop Solution was added and tapped. An Emax Plus microplate reader was used with Soft Max Pro7 (version 7.0.2; Molecular Devices,USA). To remove the effects of well uniformity and well flaws, we subtracted the readings at 570 nm from the readings at 450 nm.

### Statistical analysis

Statistical analyses were performed using R (2023.03.0 + 386). Wilcoxon rank sum test was used for paired analyses and Fisher’s exact test was used to test for gender differences among the study participants. The percentages of target protein-expressing cells were obtained under antigen- stimulated conditions minus those obtained under unstimulated conditions.

## Results

### Characteristics of study participants

We conducted flow cytometry analysis on groups of 15 participants each, and among these participants, TB (n=6) and LTBI (n=12), where cell numbers were adequate, had a paired ELISA run. Additionally, ELISA for IL-10 and IFN-γ was extended to TB (n=20) and LTBI (n=15) participants from the same cohort. For IL-17 ELISA, analysis was performed on samples collected from a separate culture, TB (n=12) and LTBI (n=11), as it required 100μl/well of cell culture supernatant. The subjects analyzed for IL-17 did not overlap with those analyzed using flow cytometry (**Supplementary Figure 2**). Results from the statistical analyses revealed no notable disparities in age and gender between the two groups of participants, both in the flow cytometry and ELISA analyses. Active TB (n=15) and LTBI (n=15) were analyzed using flow cytometry, (**Table 1**). None of the participants in either group reported a history of alcohol consumption or diabetes. The BMI median and interquartile range (IQR) for each group were (18.3 [16.5- 19]) and (22.3 [18.65-27.1]), respectively (p=0.005, Wilcoxon rank sum test). 33.3% and 0% of the individuals had a documented history of either current or past smoking, respectively (p=0.042, Fisher’s exact test). A total of 23 subjects for IL-17 ELISA were included: active TB (n=12) and LTBI (n=12) (**Table 2A**). There were no significant differences in BMI, past alcohol consumption, or diabetes history between the two groups. A documented history of either current or past smoking was observed in 36.3% and 9% of individuals, respectively (p=0.0459, Fisher’s exact test). A total of 53 subjects were includes for IFN-γ and IL-10 ELISA assays active TB (n=26) and LTBI (n=27) (**Table 2B**). There were no significant differences in past alcohol consumption and diabetes history between the two groups. The BMI (kg/m2) median, and interquartile range (IQR) for each group were (18.3[16.5-19.4]) and (21.9[18.8-24.8]), respectively (*p*=0.003178, Wilcoxon rank sum test). 24% and 3.7% of individuals had a documented history of either current or past smoking, respectively (p=0.0459, Fisher’s exact test).

**Table 1:** Characteristics of participants included in the flow cytometry analysis.

			TB (n = 15)	LTBI (n = 15)
Age (Years)	Median (IQR) Mean (SD)		23.0 (20 - 33.5) 27.47 (10.20)	22.0 (17.5 – 30.5) 26.00 (12.60)
Gender	n (%)	Male	9 (60.0%)	7 (46.7%)
		Female	6 (40.0%)	8 (53.3%)
BMI (kg/m^2^)	Median (IQR) Mean (SD)		18.3 (16.5- 19) 17.82 (1.66)	22.3 (18.65-27.1) 23.32 (5.81)
Smoke	n (%)	Current/Past	33.3	0
		Never	66.7	100
Alcohol	n (%)	Current/Past	0	0
		Never	100	100
Diabetes	n (%)	Yes	0	0

TB, tuberculosis; LTBI, latent TB infection; IQR, interquartile range; SD, standard deviation.

**Table 2A:** Characteristics of participants included in the ELISA analysis (IL-17).

IL-17			TB (n = 12)	LTBI (n = 11)
Age (Years)	Median (IQR) Mean (SD)		42.5 (23.25 - 50) 37.2 (13.4)	27 (23 – 40.5) 33.1 (15.5)
Gender	n (%)	Male	9 (75.0%)	4 (36.3%)
		Female	3 (25.0%)	7 (63.6%)
BMI (kg/m^2^)	Median (IQR) Mean (SD)		19.1 (17.75-22.3) 20.81 (4.46)	20.3 (18.8–23.35) 21.27 (3.44)
Smoke	n (%)	Current/Past	36.3	9
		Never	63.7	91
Alcohol	n (%)	Current/Past	0	9
		Never	100	91
Diabetes	n (%)	Yes	18.18	0

TB, tuberculosis; LTBI, latent TB infection; IQR, interquartile range; SD, standard deviation.

**Table 2B:** Characteristics of participants included in the ELISA analysis (IFN-γ, IL-10).

IFN-γ IL-10			TB (n = 26)	LTBI (n = 27)
Age (Years)	Median (IQR) Mean (SD)		30 (21 - 45) 32.03 (12.9)	23 (18 – 35) 27.92 (13.5)
Gender	n (%)	Male	18 (66.7%)	12 (44.4%)
		Female	8 (33.3%)	15 (55.6%)
BMI (kg/m^2^)	Median (IQR) Mean (SD)		18.3 (16.5-19.4) 18.9 (3.6)	21.9 (18.8-24.8) 21.8 (3.8)
Smoke	n (%)	Current/Past	24	3.7
		Never	76	96.3
Alcohol	n (%)	Current/Past	0	3.7
		No	100	96.3
Diabetes	n (%)	Yes	8	0

TB, tuberculosis; LTBI, latent TB infection; IQR, interquartile range; SD, standard deviation.

### Flow cytometry analysis of cytokine+ CD4+ T cells following M.tb antigen stimulation

**Figure 1:**
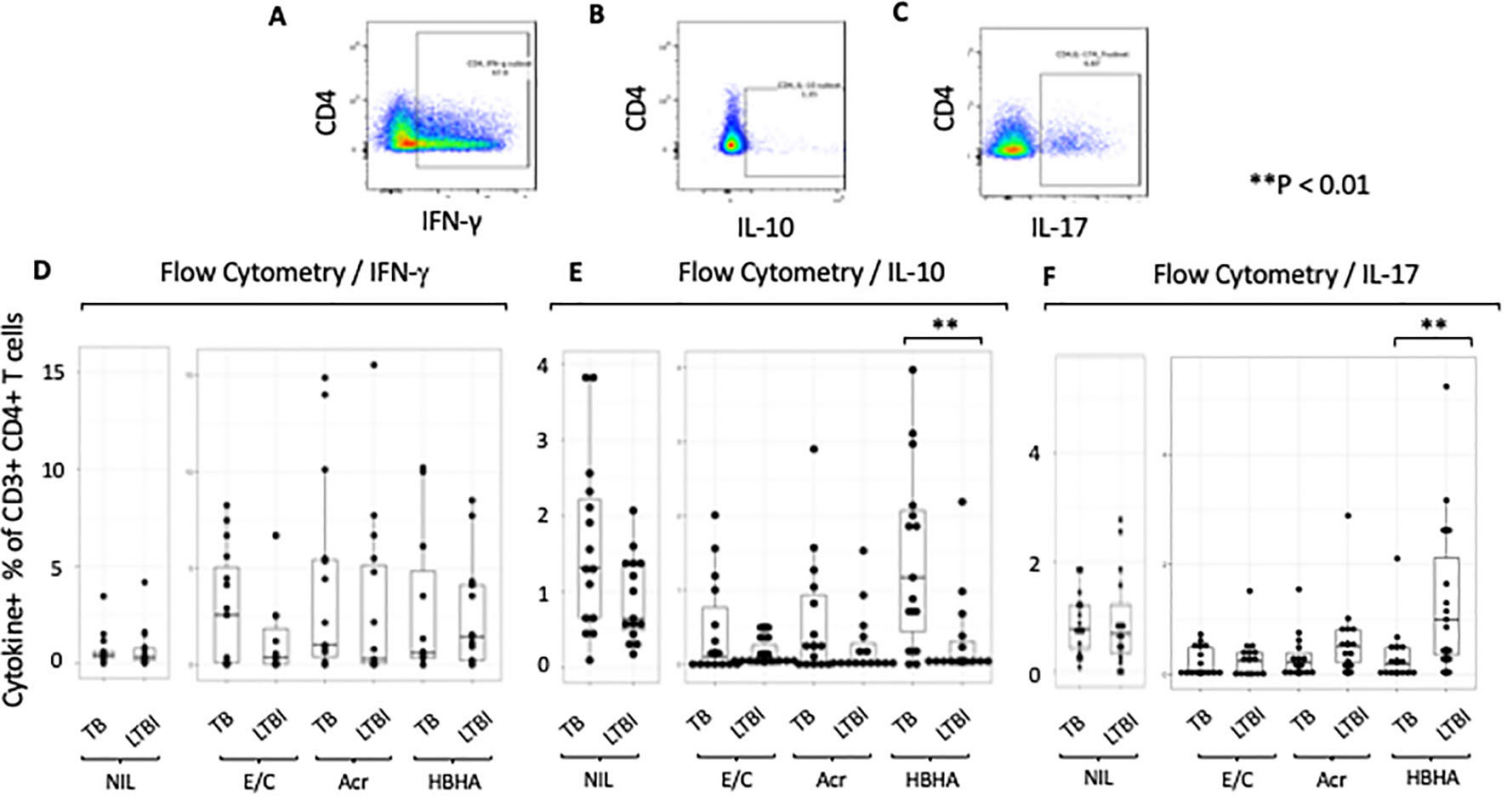
Characteristics of cytokine responses during tuberculosis antigen stimulation in the TB and LTBI groups with flowcytometry. PBMCs isolated from subjects with TB or LTBI were stimulated with ESAT-6/CFP-10, Acr or HBHA. The percentages were obtained under stimulated conditions minus those obtained under unstimulated conditions for controls. **(A–C)** Representative FACS plots of IFN-γ **(A)**, IL-10 **(B)**, and IL-17 **(C)** expression in CD4+ cells stimulated with PMA. **(D–F)** Box plot with dot plot shows the concentrations of cytokines following each antigen stimulation of PBMC in TB (n=15) or LTBI (n=15) groups analyzed by flow cytometry. IFN-γ for **(D)**, IL-10 for **(E)**, IL-17 for **(F)**. Statistical significance was determined using the Wilcoxon rank sum test. **p < 0.01.

We first compared the ratios of antigen-specific cytokine-producing CD4+ cells between the groups. A representative plot is shown (**Figures 1A–C**). We found no differences in the percentage of IFN-γ+CD4+ T cells between TB and LTBI after stimulation with M.tb antigens (**Figure 1D**). However, following HBHA stimulation, there was a significantly higher %CD4+ IL-10+T cells in active TB than in LTBI (*p*=0.0038), but not following E/C or Acr stimulation (**Figure 1E**). In contrast, following HBHA stimulation, the proportion of IL-17+ CD4+ T cells was significantly increased in participants with LTBI compared to those with active TB (median [IQR] 0.64 [0.147-1.223] for TB and 1.00 [0.355-2.12]) for LTBI; *p*=0.0076; **Figure 1F**). The results of the flow cytometry experiments are summarized in **Table 3**.

**Table 3:** Comparison of cytokine production in TB and LTBI.

Method	Antigen	IFN-γ	IL-10	IL-17
ELISA	E/C	No difference	TB *	No difference
	Acr	LTBI *	TB *	LTBI *
	HBHA	LTBI **	TB *	No difference
Flow cytometer	E/C	No difference	TB*	No difference
	Acr	No difference	TB*	No difference
	HBHA	No difference	TB **	LTBI **

The TB category mentioned is the category with the highest value.The Kruskal–Wallis test with Wilcoxon rank sum test was used to compare the groups. (*P < 0.05, **P < 0.01).

### Analysis of memory T cell subsets following M.tb antigen stimulation

**Figure 2:**
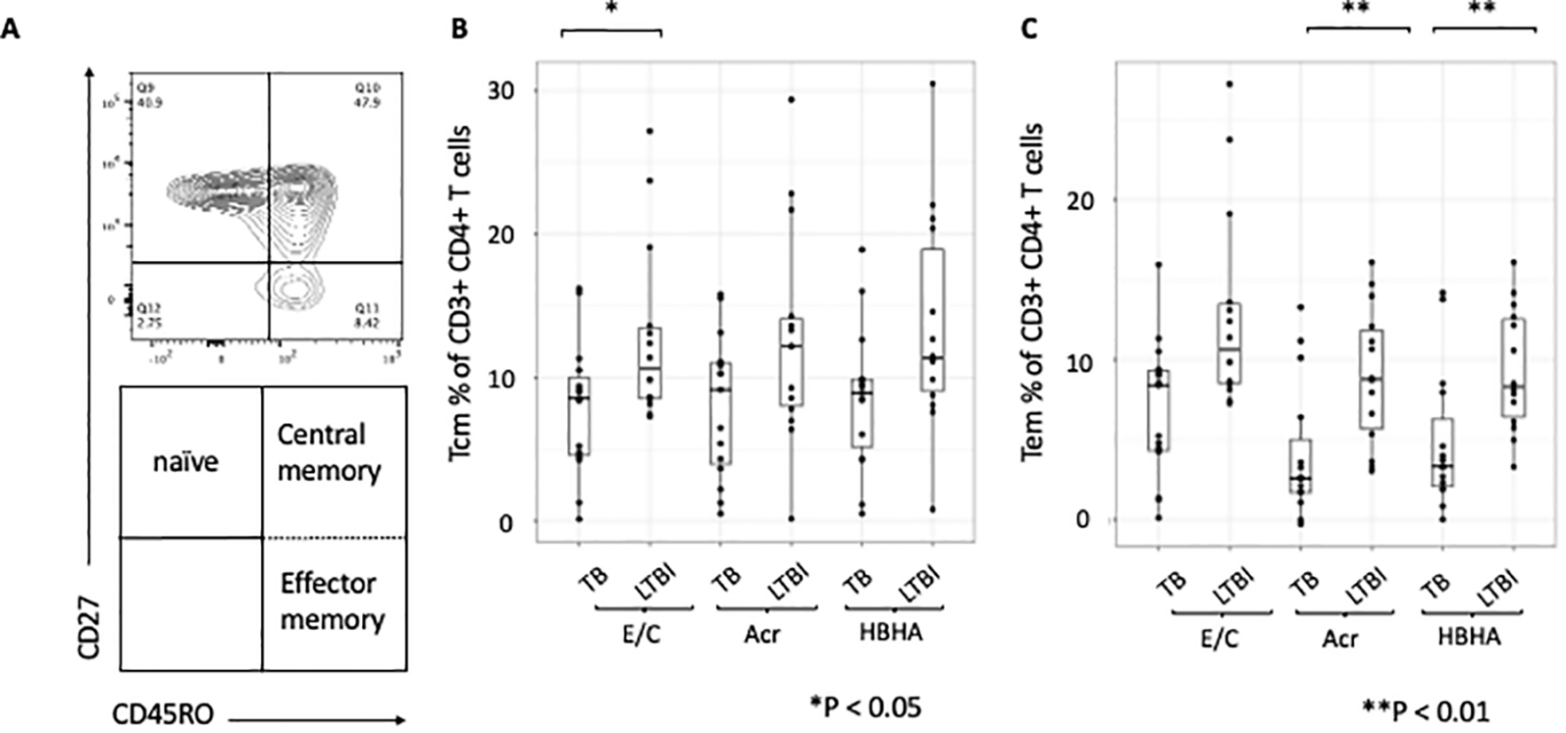
Differentiation of memory T cells stimulated by various tuberculosis antigens. Statistical significance was determined by Wilcoxon rank sum test. The percentages of antigen-specific Tcm and Tem were obtained by subtracting the baseline values of NIL samples. **(A)** Contour and dot plots from flow cytometry analysis representing memory T cells (Tm) among CD3+CD4+ cells. Tm cells (right box) are defined as central memory T cells (Tcm, CD27+CD45RO+ cells) and effector memory T cells (Tem, CD27-CD45RO+ cells). **(B)** Box plot with dot plot shows the frequencies of Tcm in CD3+CD4+ T cells following antigen stimulation of PBMC in TB (n=15) and LTBI (n=15) groups. **(C)** Box plot with dot plot shows the frequencies of Tem in CD3+CD4+ T cells following antigen stimulation of PBMC in TB (n=15) or LTBI (n=15) groups.

We next compared proportion of memory T-cell subsets between participants with active TB and those with LTBI following Ag stimulation. The gating strategy is shown (**Supplementary Figure 3**). We gated on central memory CD4+ T cells (Tcm) identified as CD27+CD45RO+ and effector memory CD4+ T cells (Tem) identified as CD27-CD45RO+ (**Figure 2A**). The proportions of Tcm and Tem in absence of any stimulation were not significant (p=0.48 and p=0.085, respectively; **Supplementary Figures 4A**, **B**). However, the proportion of Tcm was higher in participants with LTBI than those with active TB following E/C stimulation (p=0.041) (**Figure 2B**), while the proportion of Tem was higher in participants with LTBI than those with active TB following both Acr and HBHA stimulation (p < 0.01 for both; **Figure 2C**).

### Analysis of cytokine production from memory CD4+T cells following M.tb antigen stimulation

**Figure 3:**
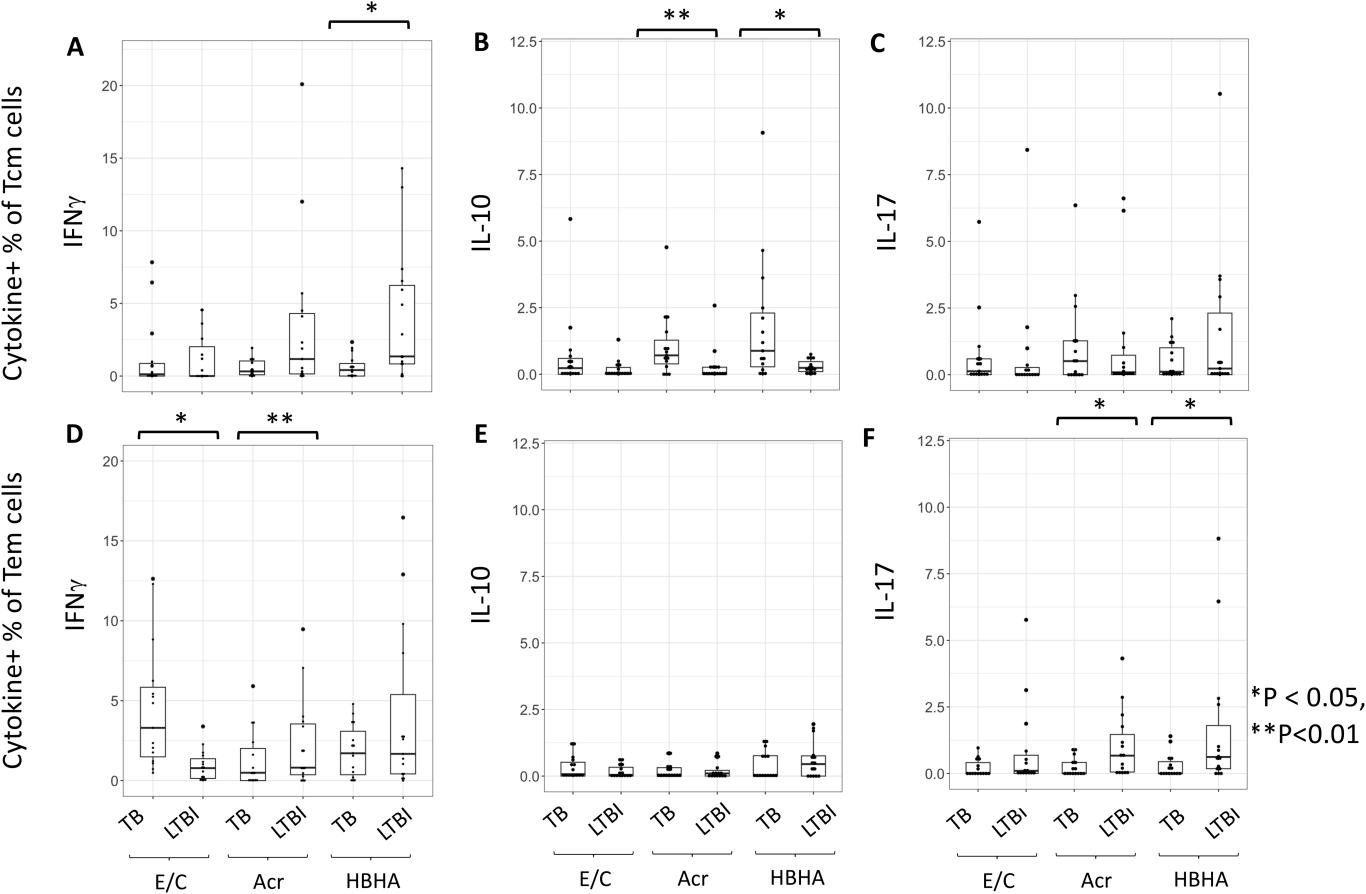
The proportion of cytokine-expressing cells in Tcm and Tem subsets. **(A–F)** Frequencies of background-subtracted M.tb antigen-specific cytokine-expressing cells within Tcm (Central Memory T cells) for **(A–C)** and Tem (Effector Memory T cells) for **(D–F)** subsets. This figure illustrates the distribution of IFN-γ **(A, D)**, IL-10 **(B, E)**, and IL-17 **(C, F)** expressing cells in response to Mycobacterium tuberculosis antigen stimulation. PBMC collected from TB (n=15) and LTBI (n=15) groups were analyzed by flow cytometry. Statistical significance was determined by Wilcoxon rank sum test. *p < 0.05, **p < 0.01.

We next measured M.tb antigen-specific cytokine production within the different subsets of memory CD4+ T cells. The proportion of HBHA-induced IFN-γ+ Tcm cells was significantly higher in individuals with LTBI than in those with active TB (p < 0.01) (**Figure 3A**). Conversely, the proportion of Acr- and HBHA-induced IL-10+ Tcm cells were significantly higher in individuals with active TB than in those with LTBI (p < 0.01 and p < 0.05, respectively) (**Figure 3B**). Following E/C stimulation, no difference was observed in the percentage of cytokine-producing Tcm cells between participants with active TB and LTBI. We also found no difference in the percentage of IL-17+ Tcm cells between participants with active TB and LTBI following E/C, Acr or HBHA stimulation (**Figure 3C**). Among effector memory T cells (Tem), the proportion of Acr-induced IFN-γ + Tem cells was significantly higher in participants with LTBI than those with active TB (P<0.001) (**Figure 3D**). Similarly, the proportion of Acr- and HBHA-induced IL-17+ Tem cells was higher in the LTBI group compared to active TB group (p<0.05) (**Figure 3F**). In contrast, the proportion of E/C-induced IFN-γ + Tem cells was higher in participants with active TB compared to LTBI (p<0.05) (**Figure 3D**). We found no difference in the percentage of IL-10 + Tem cells between active TB and LTBI following M.tb antigen stimulation (**Figure 3E**).

### Characterization of Treg and cytokine-producing cells following HBHA stimulation

**Figure 4:**
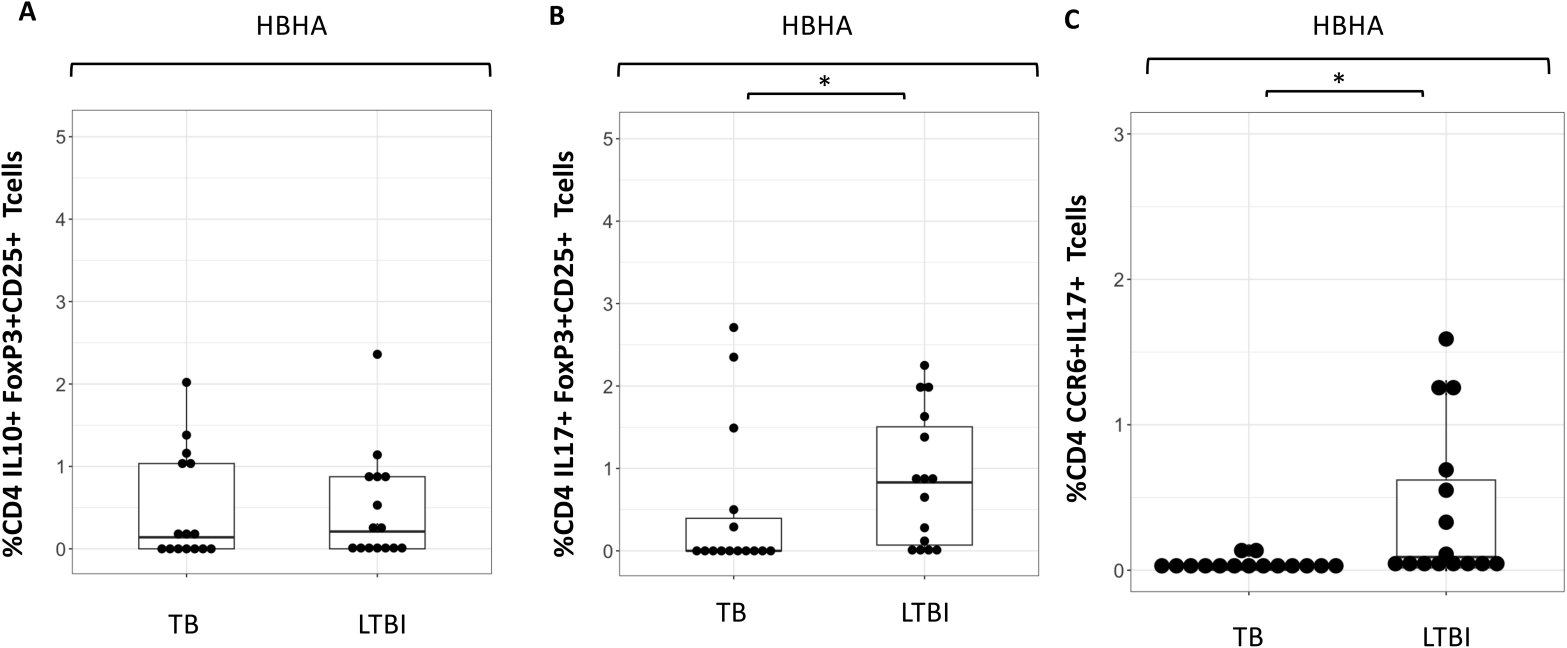
Characteristics of cytokine expressing cells during HBHA stimulation in the TB and LTBI groups with flowcytometry. Boxplot overlaid with dot plot illustrating the distribution of cytokine expressing cells following with HBHA stimulation in the TB and LTBI groups. **(A)** IL-10+FoxP3+CD25+ CD4 Tcells, **(B)** IL-17+FoxP3+CD25+ CD4+T cells and **(C)** CCR6+IL-17+ CD4+T cells. PBMC in TB (n=15) or LTBI (n=15) group was analyzed by flow cytometry.Statistical significance was determined by Wilcoxon rank sum test. *p < 0.05.

Despite differences in CD4+IL10+ cells, no difference in %CD4+ FoxP3+CD25+cells were seen following any of the stimulation conditions (**Supplementary Figures 5A, B**). However, the proportion of IL-10+CD4+ T cells was higher following HBHA stimulation in participants with active TB compared to those with LTBI. We therefore compared the expression of these cytokines in HBHA-stimulated FoxP3+CD25+CD4+ T cells and CCR6+CD4+ T cells. No difference in the proportion of IL-10+FoxP3+CD25+CD4+ T cells was observed in the HBHA-stimulated samples (**Figure 4A**). In contrast, IL-17+FoxP3+CD25+ cells and IL-17+CCR6+ cells induced by HBHA stimulation were significantly higher in participants with LTBI (**Figures 4B**, **C**), (p<0.05 and p<0.05, respectively).

### Analysis of ‘IFN-γ and IL-10’ or ‘IL-17 and IFN-γ’ double cytokine-positive CD4+ T cells

**Figure 5:**
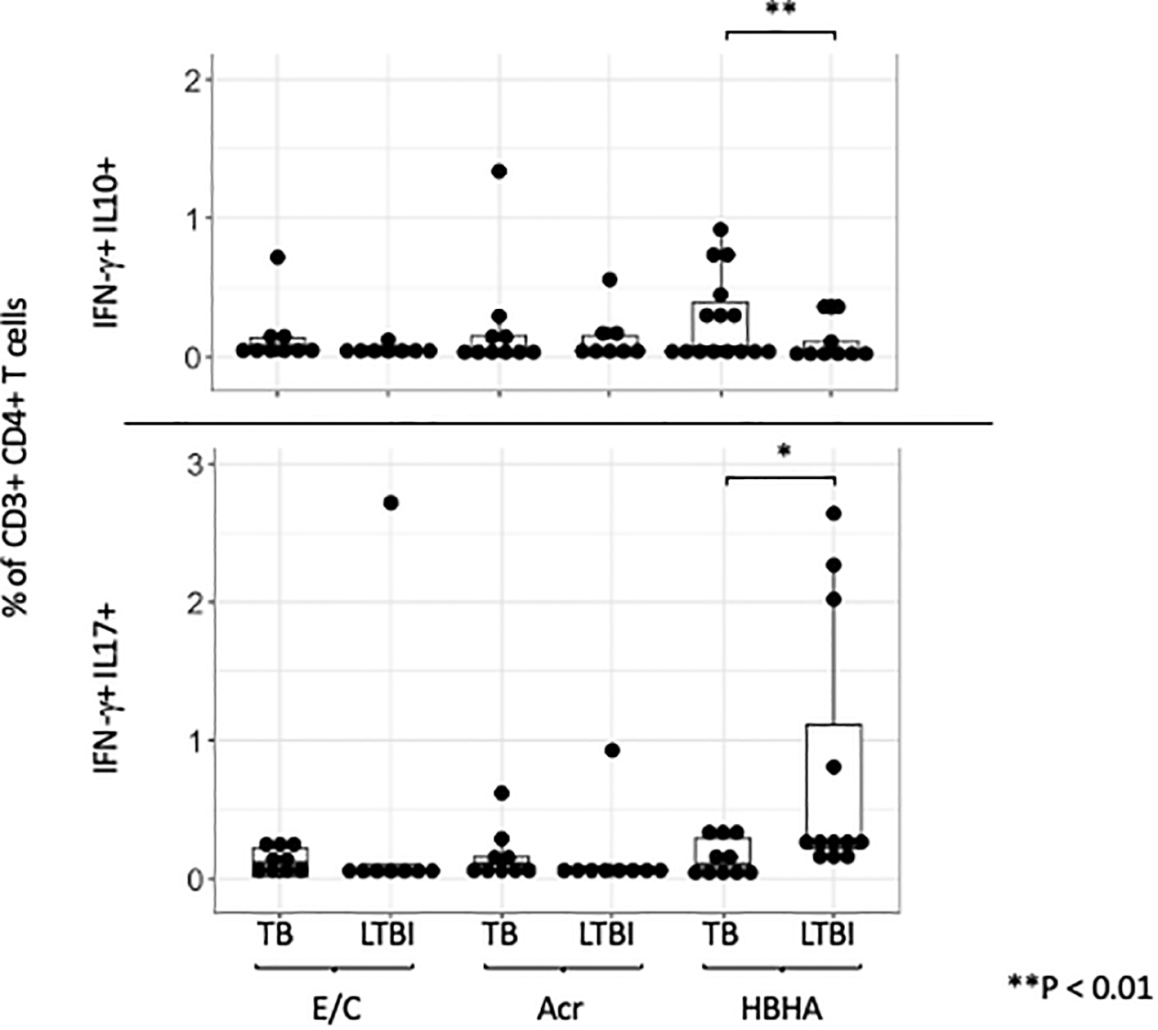
IFN-γ and IL-10/IL-17 double positive cells across different M.tb antigens. Boxplot overlaid with a dot plot showing the distribution of double-positive cells expressing both IFN-γ and IL-10 (above)/IFN-γ and IL-17 (below) in CD4+ T cells across different antigens (E/C, Acr and HBHA). The Y-axis is displayed on a logarithmic scale to accommodate a wide range of values, with the minimum value set to 0.1. PBMC in TB (n=15) or LTBI (n=15) group was analyzed by flow cytometry.Statistical significance was determined by Wilcoxon rank sum test. *p < 0.05, **p < 0.01.

Double-cytokine positive CD4+ T cells, including those that produce both ‘IFN-γ and IL-10’ or ‘IL-17 and IFN-γ’, play a critical role in the immune response to chronic infectious diseases. We therefore investigated if any of our M.tb antigens induced these cells using flow cytometry. A representative plot is shown (**Supplementary Figure 6**). The frequencies of E/C and Acr-specific ‘IFN-γ and IL-10’ and ‘IFN-γ and IL-17’ double-positive CD4+T cells were not significantly different between the two groups (**Figure 5**). However, we observed higher HBHA-specific ‘IFN-γ and IL-10’ double-positive CD4+ T cells in participants with active TB compared to those with LTBI (p= 0.036). In contrast, HBHA-specific ‘IFN-γ and IL-17’ double-positive CD4+ T cells were significantly higher in LTBI compared to active TB participants (p<0.05) (**Figure 5**).

### ELISA analysis of supernatants following M.tb antigen stimulation

Our flow cytometry results showed that the latency-associated antigen ‘HBHA’ induced the pro- inflammatory cytokine IFN-γ in LTBI participants, whereas it induced the anti-inflammatory cytokine IL-10 in active TB participants. We therefore conducted ELISA to verify whether this result could be used in a point-of-care (POC) test to differentiate between active and latent tuberculosis. We measured both IFN-γ and IL-10 levels with ELISA in 26 tuberculosis patients and 28 latent tuberculosis subjects. Since there were sufficient cell numbers, 6 tuberculosis patients and 12 latent tuberculosis patients had paired analysis of their samples by ELISA and flow cytometry study (**Supplementary Figure 2**).

**Figure 6:**
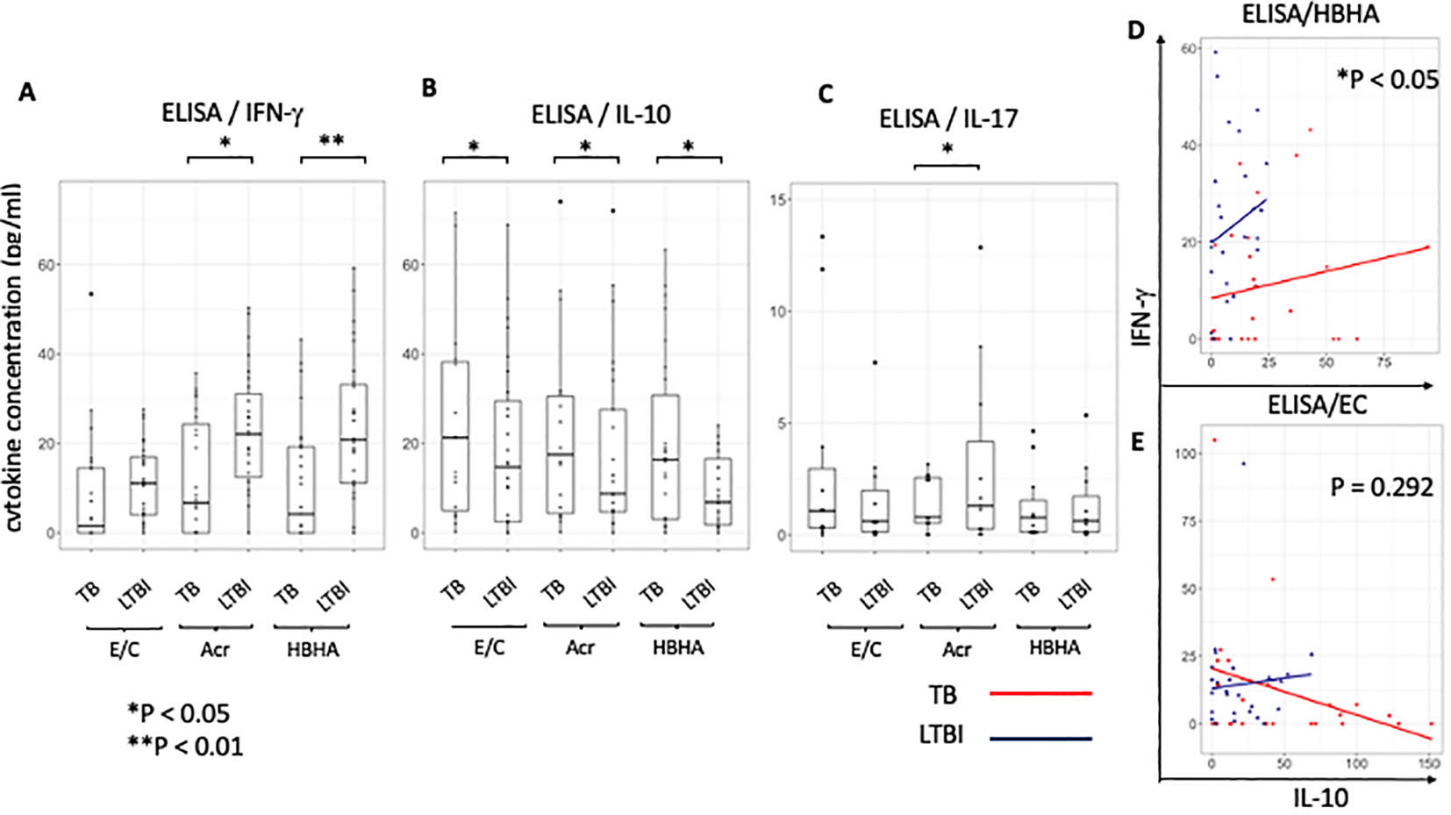
Characteristics of cytokine responses during tuberculosis antigen stimulation in the TB and LTBI groups with ELISA. **(A–C)** Box plot with dot plot shows the cytokine following each antigen stimulation to PBMC in TB (n=26) or LTBI (n=27) group analysed by ELISA.IFN-γ for **(A)**, IL-10 for **(B)**, IL-17 for **(C)**. **(D, E)** Scatter plot depicting the relationship between IL-10 and IFN-γ levels induced with HBHA **(D)** and ESAT-6/CFP-10 **(E)** stimulation for categories ‘TB’ (red) and ‘LTBI’ (blue). Each point represents a data point within a category.Y-axis represents IFN-γ levels and X-axis represents IL-10 levels. The plot includes regression lines fitted with a linear model that accounts for the interaction term between the cytokines. P-values indicate the significance of the interaction effect between the two cytokines (HBHA,p=0.020; ESAT-6/CFP-10,p=0.292). Points are colored and shaped according to their respective categories (TB; Red circle, LTBI; Blue triangle). Statistical significance was determined by Wilcoxon rank sum test. *p < 0.05, **p < 0.01.

Our results following ELISA showed no significant difference in IFN-γ production following E/C stimulation between TB and LTBI groups (**Figure 6A**). However, following Acr or HBHA stimulation, IFN-γ levels in the LTBI group were significantly higher than those in the active TB group (p < 0.05, p<0.01 respectively; **Figure 6A**). In contrast, IL-10 levels were significantly higher in participants with active TB compared to those with LTBI following stimulation with all antigens (p< 0.05; **Figure 6B**). We analyzed samples from an additional 11 active TB patients and 12 LTBI participants who did not overlap with the flow cytometry results using ELISA for detection of IL-17. Acr-induced IL-17 production was higher in participants with LTBI compared to those with active TB (median [IQR] for active TB (1.00 [0.53-2.50]) and 1.30 [0.26-4.17] for LTBI; p<0.0001; **Figure 6C**) whereas no difference was observed following E/C or HBHA stimulation (**Figure 6C**). However, the measured values were generally low, making them likely unsuitable for POC testing. ELISA results are summarized in **Table 3**.

To ascertain the balance of cytokine production at the individual level, the relationship between pro-inflammatory cytokine ‘IFN-γ’ and anti-inflammatory cytokine ‘IL-10’ in individuals was analyzed using a scatter plot between the active TB (n=26) and LTBI (n=27) groups and statistically compared using the interaction term in a linear model. We found a significant difference in the relationship between IL-10 and IFN-γ levels between the two groups following stimulation with latency-associated antigens. Upon HBHA stimulation, as IL-10 production increased, IFN-γ production increased. The ratio of the increase in IFN-γ to the increase in IL-10 was significantly greater in the LTBI group (HBHA: P<0.05; **Figure 6D**). In active TB patients, some participants had elevated IFN-γ with low IL-10 levels. Additionally, a minority of cases exhibited high levels of both the cytokines. In contrast, upon EC stimulation in the active TB group, IFN-γ production decreased as IL-10 production increased but this was not significant (ESAT-6/CPF-10: p=0.292; **Figure 6E**). Similar to HBHA, Acr stimulation resulted in significant differences in the interaction plot of pro-inflammatory cytokines versus anti-inflammatory cytokines (ie IFN-γ versus IL-10 levels) (p=0.013; **Supplementary Figure 7A**). This was also true for the IL-17 and IL-10 levels (p=0.020; **Supplementary Figure 7B**). However, the interaction plot of IFN-γ vs. IL-17 levels (**Supplementary Figure 7C**) did not show a significant difference.

## Discussion

This study investigated immune responses in active TB and LTBI participants from a TB-endemic setting, highlighting the potential of measuring CD4+ T cell production of IFN-γ, IL-10, and IL-17 after stimulation with latency-associated antigens, particularly HBHA, to differentiate between active TB and LTBI.

Conventional IGRA fails to distinguish active TB from LTBI via IFN-γ responses, likely due to the antigens used and the cytokines detected (38). Our ELISA results showed higher IFN-γ levels in LTBI than in active TB after stimulation with latency antigens, consistent with findings from other regions and ethnic groups (14–18). Flow cytometric analysis showed no differences in IFN-γ production by CD4+ T cells (**Figure 1D**), in contrast to the ELISA results, possibly because of the IFN-γ+ percentage of non-CD4+ T cells after Acr and HBHA stimulation. Although CD8+ T cells respond to HBHA, they are not the primary IFN-γ producers (18, 39). IFN-γ production from CD3+CD4-CD8- lymphocytes upon HBHA stimulation has also been reported (40), necessitating further investigation into of the phenotype of these cells. Previous studies in Japan found higher CD4+ IL-10+ T cell percentages in patients with TB than in healthy controls after Acr stimulation (23). Similarly, ELISA data from this study showed elevated IL-10 levels in active TB group compared to LTBI group under all antigen stimulation conditions in The Gambia (**Figure 6B**), indicating that IL-10 response to latency antigens is independent of TB prevalence and host genetics. However, unlike the Japanese study, this present study found significant differences in HBHA-induced CD4+IL-10+ T cell expression, suggesting potential ethnic differences in the antigen specificity. Combining IL-10 and IFN-γ may differentiate active from latent TB using ELISA or lateral flow tests. Significant differences in the relationship between pro- and anti-inflammatory cytokines were observed between the latency-associated and active TB antigens. The cytokine production balance during latency-associated antigen stimulation indicated that individuals with higher levels of inflammatory cytokines exhibited lower level of inhibitory cytokines, and *vice versa*. Regression lines differed significantly between the two groups. However, cytokine production following stimulation with E/C showed no significant differences. At the individual level, the balance between anti-inflammatory and pro-inflammatory cytokines against latency-associated antigens may be pathogenic.

Research suggests that active TB is associated with higher effector memory to central memory Th1 cell ratios than LTBI (26), with ESAT-6-specific Tcm more prevalent in active TB and Tem predominating in LTBI (27). In contrast, our results showed no difference in Tcm or Tem between TB and LTBI without stimulation, and higher Tcm in LTBI than TB with E/C stimulation possibly because LTBI is detected in household contacts (HHC) constantly exposed to Mtb. In long-term LTBI, effector memory or effector CD8 T cells were highly prevalent following DosR antigen stimulation (26, 41). We observed that effector memory CD4+ T cells were significantly higher in LTBI than in active TB following both Acr and HBHA stimulation. This suggests persistent latency-associated antigen exposure in LTBI individuals.

TB is a chronic disease with a long period of exposure to latency-antigens. IL-10 has been implicated in the ability of M.tb to evade immune responses and mediate long-term infection owing to its anti-inflammatory role (42). Indeed, we found that Tcm produced higher levels of IFN- γ and IL-10 following stimulation with latency-associated antigens in active TB patients compared to LTBI. This suggests that latency-associated antigens induce opposite effects on inflammatory cytokines depending on the disease state, particularly in Tcm and this may define pathogenesis. In Tem, latency-associated antigens also induced higher levels of IFN-γ and IL-17 in LTBI individuals. These results indicate the presence of a persistent immune response against latency-associated TB antigens. Therefore, it should be noted that vaccines and adjuvants containing latency-associated antigens such as HBHA may induce stronger inflammation in LTBI subjects than in TB patients. Additionally, we found that LTBI subjects had a significantly higher proportion of Th17 cells producing IL-17 than active TB patients (34) consistent with previous studies (11–15).

We found higher levels of HBHA induced Th17 (CCR6+) production from CD4+ T cells in LTBI participants. IL-17A is involved in mature granuloma formation in mycobacteria-infected lung (43). Therefore, in patients with latent TB, the response to latent TB antigens may be stronger than that to active TB antigens to prevent progression to TB by granuloma cell formation. In contrast, IL-10-producing CD4 T cells were induced in active TB patients following latency-antigen stimulation. IL-10 is produced by regulatory T cell subsets such as IL-10-producing type 1 regulatory T (Tr1) cells (44), which express IL-10 and IFN-γ. Our results also showed that HBHA induced IL-10+IFN-γ+CD4+ T cells in active TB. This cytokine profile, unique to Tr1 cells, allows them to regulate immune responses by suppressing inflammation and promoting immune functions (45, 46). It is possible that the active TB disease developed because of suppression of inflammation against Mtb infection due to induction of IL-10+IFN-γ+CD4+T cells.

Recently, IL-17-producing FoxP3+CD25+Tregs were discovered (47). These subsets are commonly believed to serve as intermediaries during the transition of Tregs to Th17 cells (48). Our results showed that IL-17+ FoxP3+CD25+Treg CD4 T cells were induced by HBHA in LTBI subjects. We found no significant differences in the % of FoxP3+CD25+Treg CD4 T cells in LTBI compared to active TB, in contrast to findings from previous studies (49, 50), which reported higher FoxP3+CD25+Treg cell frequencies in active TB than in LTBI. Several factors might explain this discrepancy. Our LTBI subjects were household contacts of tuberculosis patients, with potentially higher exposure levels, and a more heterogeneous population, possibly including individuals with subclinical TB or those on the verge of developing active TB. The limitations of this study was the relatively small sample size: 15 patients each for active TB and LTBI in flow cytometry, and 26 for active TB and 27 for LTBI in ELISA, reducing the generalizability of the findings. Including a larger and more diverse patient population with varying TB severities and demographic backgrounds would strengthen the study. In addition, paired analysis of flow cytometry and ELISA was only performed in a small proportion of participants.

IL-17 levels measured by ELISA were low, possibly due to using frozen cells. Therefore, a more sensitive assay system is required for clinical application. Flow cytometry is challenging in countries with high TB endemic rates because of high technology and costs. However, combining IL-10 and IFN-γ ELISA with latency antigens could be applicable for point-of-care tests. Future plans include comparing disease-progressing and non-progressing subjects using longitudinal cohorts at the single-cell level to observe the time course and plasticity of cytokine-expressing cell differentiation. Assessing additional cytokines such as TNF and IL-22 is also important. Longitudinal data could offer valuable insights into TB infection dynamics and progression. This could lead to the development of novel therapeutics and diagnostics.

## Data Availability

The raw data supporting the conclusions of this article will be made available by the authors, without undue reservation.

## Supplementary material

Supplementary Figure 1 Conditions set for antigen concentration in measuring cytokine-expressing CD4 T cells. PBMCs (TB=2), at a concentration of 5×10^5 cells were stimulated with various antigen concentrations. The number of cytokine-positive cells was measured by flow cytometry.

Supplementary Figure 2 Study design. Participants were selected based on peripheral blood mononuclear cell sample availability. **(A)** Flow cytometry (TB:n=15,LTBI:n=15)and IFN-γ, IL-10 ELISA (TB:n=26,LTBI:n=27). **(B)** IL-17 ELISA(TB:n=11,LTBI:n=12).

Supplementary Figure 3 Representative dot plots showing the gating strategy of CD3+CD4+ T cell subsets from fresh PBMC from healthy controls. Outline of the standard gating strategy for analyzing samples. Example data are typical for fresh PBMC from healthy controls incubated with PMA for 6 hours. As shown in the first plot, the cells were FSC-A vs. FSC-H to gate out doublets. In the next plot, cells were FSC-A vs. SSC-A to gate the lymphocytes. The last plot shows gating for live cells.

Supplementary Figure 4 Tcm or Tem cells of CD4+Tcells. **(A)** Box plots with dot plot show the frequencies of Tcm in CD3+CD4+ T cells in the absence of any *in vitro* stimulation of PBMC in TB (n=15) and LTBI (n=15) group. Statistical significance was determined by Wilcoxon rank sum test. **(B)** Box plots with dot plot show the frequencies of Tem in CD3+CD4+ T cells in the absence of any *in vitro* stimulation of PBMC in TB (n=15) or LTBI (n=15) group. Statistical significance was determined by Wilcoxon rank sum test.

Supplementary Figure 5 Comparison of Treg (FoxP3+CD25+ CD4+ Tcell) induction by antigen stimulation between TB and LTBI groups using flow cytometry. The percentages were obtained in stimulated conditions minus those obtained in unstimulated conditions for controls. Statistical significance was determined by Wilcoxon rank sum test. **(A)** Flow cytometry plot of FoxP3+ and CD25+ in CD3+CD4+ T cells. **(B)** Box plots with dot plots show the frequencies of FoxP3+CD25+cells in CD3+CD4+ T cells stimulated with each antigen in TB and LTBI groups.

Supplementary Figure 6 Representative dot plots. Large blue dots show the IL-10+IFN-γ+CD4+Tcells or IL-17+IFN-γ+CD4+Tcells.

Supplementary Figure 7 Scatter plot of cytokine balance in ELISA following stimulation with Acr. Scatter plot of IFN-γ versus IL-10 levels **(A)**, IL-17 versus IL-10 **(B)** and IFN-γ versus IL-17 levels IFN-γ **(C)** for categories TB and LTBI. The plot includes regression lines fitted with a linear model that accounts for an interaction term between cytokines. P-values provides the significance of the interaction effect between two cytokines (**A**;p=0.013, **B**;p=0.020, **C**;p=0.515). Points are colored and shaped according to their respective categories (TB; Red circle, LTBI; Blue triangle).
