# Supplementary figures and images for: HBHA induces IL-10 from CD4+ T cells in patients with active tuberculosis but IFN-γ and IL-17 from individuals with Mycobacterium tuberculosis infection
Source: Front Immunol. 2024 Aug 27;15:1422700. doi: 10.3389/fimmu.2024.1422700 (PMC11384583; doi:10.3389/fimmu.2024.1422700)

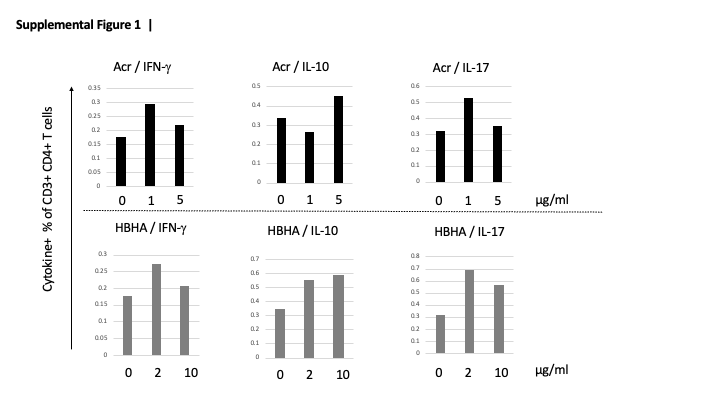

Supplement: Supplementary Figure 1 — Conditions set for antigen concentration in measuring cytokine-expressing CD4 T cells. PBMCs (TB=2), at a concentration of 5×10^5 cells were stimulated with various antigen concentrations. The number of cytokine-positive cells was measured by flow cytometry. [file Image1.tiff]

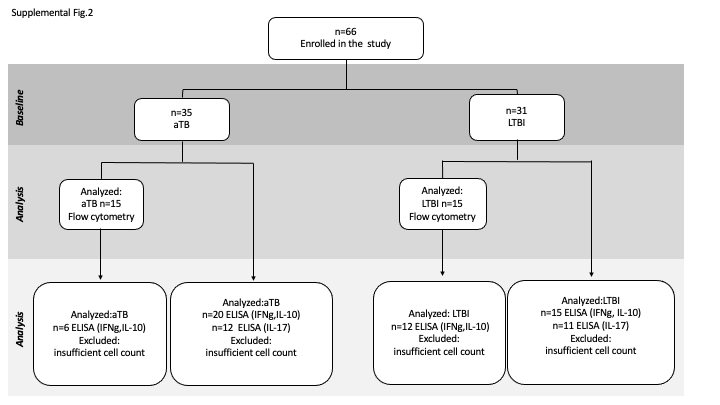

Supplement: Supplementary Figure 2 — Study design. Participants were selected based on peripheral blood mononuclear cell sample availability. (A) Flow cytometry (TB:n=15,LTBI:n=15)and IFN-γ, IL-10 ELISA (TB:n=26,LTBI:n=27). (B) IL-17 ELISA(TB:n=11,LTBI:n=12). [file Image2.tiff]

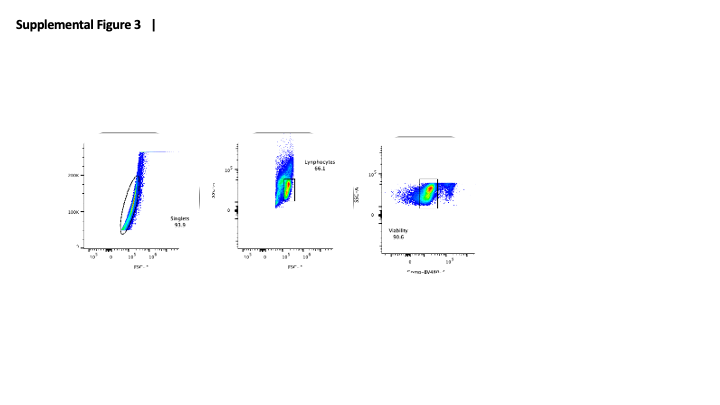

Supplement: Supplementary Figure 3 — Representative dot plots showing the gating strategy of CD3+CD4+ T cell subsets from fresh PBMC from healthy controls. Outline of the standard gating strategy for analyzing samples. Example data are typical for fresh PBMC from healthy controls incubated with PMA for 6 hours. As shown in the first plot, the cells were FSC-A vs. FSC-H to gate out doublets. In the next plot, cells were FSC-A vs. SSC-A to gate the lymphocytes. The last plot shows gating for live cells. [file Image3.tiff]

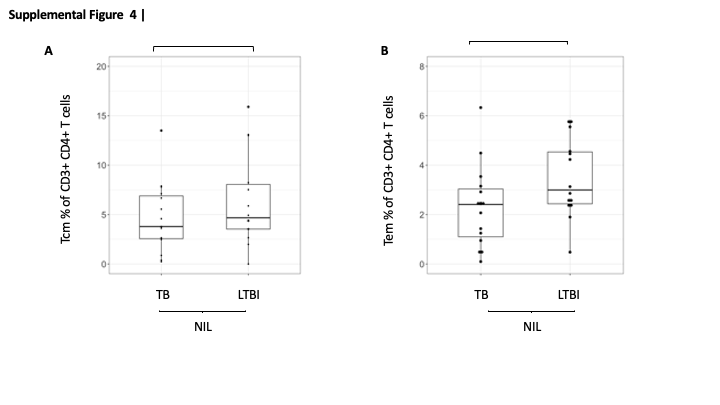

Supplement: Supplementary Figure 4 — Tcm or Tem cells of CD4+Tcells. (A) Box plots with dot plot show the frequencies of Tcm in CD3+CD4+ T cells in the absence of any in vitro stimulation of PBMC in TB (n=15) and LTBI (n=15) group. Statistical significance was determined by Wilcoxon rank sum test. (B) Box plots with dot plot show the frequencies of Tem in CD3+CD4+ T cells in the absence of any in vitro stimulation of PBMC in TB (n=15) or LTBI (n=15) group. Statistical significance was determined by Wilcoxon rank sum test. [file Image4.tiff]

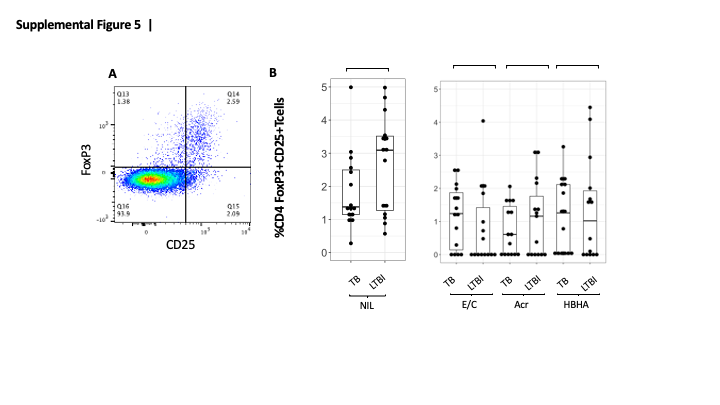

Supplement: Supplementary Figure 5 — Comparison of Treg (FoxP3+CD25+ CD4+ Tcell) induction by antigen stimulation between TB and LTBI groups using flow cytometry. The percentages were obtained in stimulated conditions minus those obtained in unstimulated conditions for controls. Statistical significance was determined by Wilcoxon rank sum test. (A) Flow cytometry plot of FoxP3+ and CD25+ in CD3+CD4+ T cells. (B) Box plots with dot plots show the frequencies of FoxP3+CD25+cells in CD3+CD4+ T cells stimulated with each antigen in TB and LTBI groups. [file Image5.tiff]

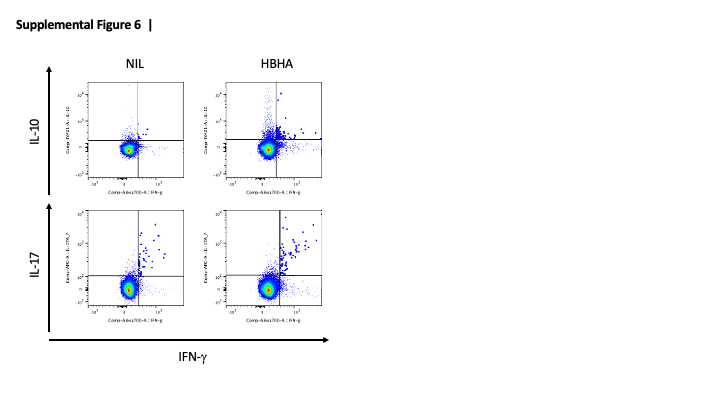

Supplement: Supplementary Figure 6 — Representative dot plots. Large blue dots show the IL-10+IFN-γ+CD4+Tcells or IL-17+IFN-γ+CD4+Tcells. [file Image6.tiff]

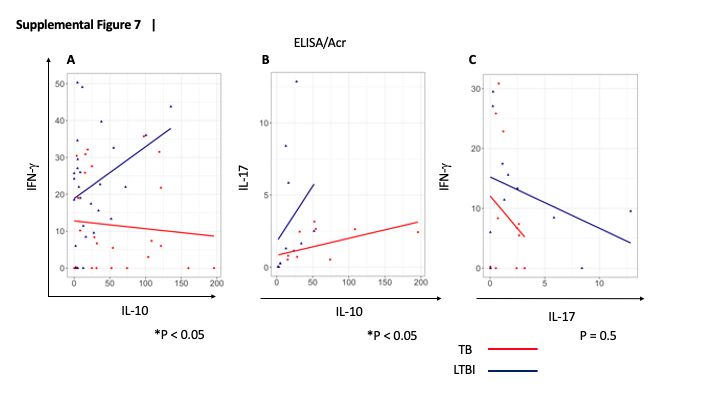

Supplement: Supplementary Figure 7 — Scatter plot of cytokine balance in ELISA following stimulation with Acr. Scatter plot of IFN-γ versus IL-10 levels (A), IL-17 versus IL-10 (B) and IFN-γ versus IL-17 levels IFN-γ (C) for categories TB and LTBI. The plot includes regression lines fitted with a linear model that accounts for an interaction term between cytokines. P-values provides the significance of the interaction effect between two cytokines (A;p=0.013, B;p=0.020, C;p=0.515). Points are colored and shaped according to their respective categories (TB; Red circle, LTBI; Blue triangle). [file Image7.tiff]
